# 

**Published:** 1890-10-25

**Authors:** 


No. 213. Vol IX.] SATURDAY, OCTOBER 25th, 1890. [ONE PENNY.

